# The Role of Podoplanin in Skin Diseases

**DOI:** 10.3390/ijms23031310

**Published:** 2022-01-24

**Authors:** Jun Asai

**Affiliations:** Department of Dermatology, Graduate School of Medical Science, Kyoto Prefectural University of Medicine, Kyoto 602-8566, Japan; jasai@koto.kpu-m.ac.jp; Tel.: +81-75-251-5586

**Keywords:** podoplanin, psoriasis, wound healing, melanoma, squamous cell carcinoma, extramammary Paget’s disease

## Abstract

Podoplanin is a sialomucin-like type I transmembrane receptor glycoprotein that is expressed specifically in lymphatic vessels, sebaceous glands, and hair follicles in normal skin. However, under pathological conditions podoplanin expression is upregulated in various cells, such as keratinocytes, fibroblasts, tumor cells, and inflammatory cells, and plays pivotal roles in different diseases. In psoriasis, podoplanin expression is induced in basal keratinocytes via the JAK-STAT pathway and contributes toward epidermal hyperproliferation. Podoplanin expression on keratinocytes can also promote IL-17 secretion from lymphocytes, promoting chronic inflammation. During wound healing, the podoplanin/CLEC-2 interaction between keratinocytes and platelets regulates re-epithelialization at the wound edge. In skin cancers, podoplanin expresses on tumor cells and promotes their migration and epithelial-mesenchymal transition, thereby accelerating invasion and metastasis. Podoplanin is also expressed in normal peritumoral cells, such as cancer-associated fibroblasts in melanoma and keratinocytes in extramammary Paget’s disease, which promote tumor progression and predict aggressive behavior and poor prognosis. This review provides an overview of our current understanding of the mechanisms via which podoplanin mediates these pathological skin conditions.

## 1. Introduction

Podoplanin, also known as gp36, T1α, D2-40, PA2.26, and aggrus, is a small sialomucin-like type I transmembrane receptor glycoprotein composed of a heavily O-glycosylated extracellular domain, a transmembrane domain, and a short intracellular domain [1,2,3]. The amino acid sequence of podoplanin is well conserved across different species [3,4] and, although originally named due to its expression in renal podocytes, podoplanin is now known to be expressed widely in different tissues and cells, including lymphatic endothelial cells, type I alveolar cells, osteocytes, osteoblasts, choroid plexus epithelial cells, mesothelial cells, glial cells, and stromal reticular cells in lymphoid organs [5]. In normal skin, podoplanin is expressed in sebaceous glands, hair follicles, and lymphatic vessels [6], where it exerts a variety of functions.

Studies in podoplanin-deficient mice have revealed that podoplanin plays important roles in lymphatic vessel formation, lung cell proliferation, alveolus formation, and myocardial differentiation during embryonic development [7,8]. After birth, podoplanin plays crucial roles in several physiological conditions, such as platelet production, immune responses, lymphangiogenesis, and hair growth [9,10]. Recent studies have shown that in various pathological skin conditions, including inflammatory diseases and cancers, podoplanin is upregulated in cells that do not normally express podoplanin and contributes toward disease development and progression [11,12,13]. In this study, we manually searched the literature available in Pubmed using the following keywords: podoplanin, skin, cutaneous, melanoma, squamous cell carcinoma, skin cancer, wound healing, psoriasis, and dermatitis. Reports that evaluated any relationship between podoplanin and a skin disease and discussed the specific roles of podoplanin in skin diseases were included in this review. We included all the reports irrespective of their year of publication.

## 2. Podoplanin Structure and Targeting Agents

**Figure 1:**
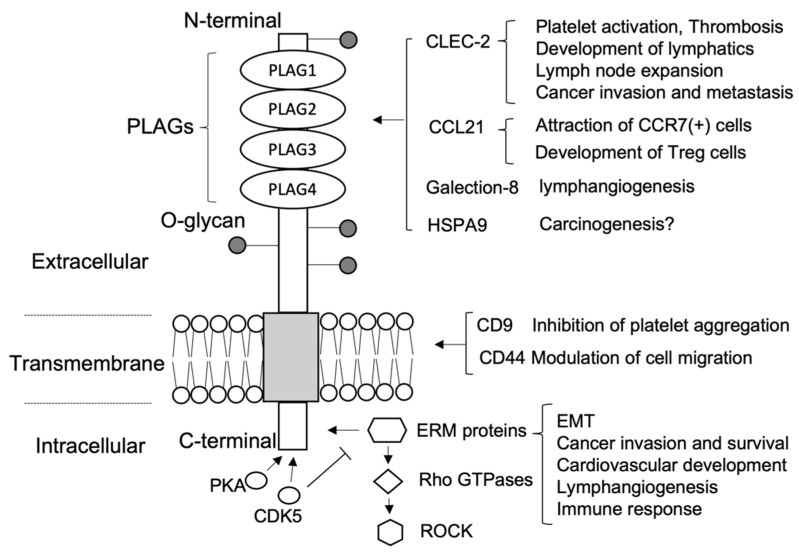
Podoplanin structure and binding partners. PLAG: platelet aggregation-stimulating domain. CLEC-2: C-type lectin-like receptor-2. HSPA9: heat shock protein A9. EMT: epithelial-mesenchymal transition. ERM: ezrin/radixin/moesin. PKA: protein kinase A. CDK5: cyclin dependent kinase 5. ROCK: Rho-associated coiled-coil kinase.

Podoplanin consists of an extracellular domain of approximately 130 amino acids, a transmembrane domain of approximately 25 amino acids, and a short intracellular domain of approximately 10 amino acids [14]. A schematic overview of the structure of podoplanin and its binding partners is shown in Figure 1.

### 2.1. Extracellular Domain

The extracellular domain of podoplanin contains four platelet aggregation-stimulating (PLAG) domains that can bind to and interact with C-type lectin-like receptor-2 (CLEC-2) expressed on platelets or hematopoietic cells, such as monocytes, dendritic cells, natural killer cells, and granulocytes [9,15,16]. Since podoplanin is not expressed in blood vessels, platelet CLEC-2 cannot bind to podoplanin under normal conditions and, thus, cannot be activated. However, under pathological conditions or during organ development, these two proteins can interact and induce platelet activation, thrombosis, lymphatic vessel development, and cancer invasion and metastasis [16,17,18]. The interaction between podoplanin and CLEC-2 positive dendritic cells has been well characterized [19,20,21]. In particular, the CLEC-2-podoplanin interaction contributes toward the intravasation of dendritic cells into lymphatic vessels and their migration to lymph nodes when the immune response is initiated.

Other proteins that bind to the extracellular domain of podoplanin include galectin-8, heat-shock protein A9 (HSPA9), and CCL21 [22,23,24,25]. Galectin-8 is a tandem-repeat type galectin that interacts with glycoproteins on the cell surface and is highly expressed on lymphatic endothelial cells. By cooperating with podoplanin, galectin-8 supports the connection between the lymphatic endothelium and the surrounding extracellular matrix [23]. Conversely, podoplanin interacts with HSPA9 on the surface of oral squamous cell carcinoma (SCC) cells. Tsuneki et al. reported the colocalization of HSPA9 and podoplanin at the periphery of oral SCC foci and, since HSPA9 was secreted from the tumor cells, their binding was thought to take place in an autocrine fashion. The authors also speculated that the HSPA9-podoplanin complex may regulate SCC cell invasion activity; however, the detailed molecular mechanisms were not evaluated [22]. CCL21 is a chemokine that is produced specifically by lymphatic endothelial cells and high endothelial venules in lymph nodes and other secondary lymphoid organs [26]. CCL21 and podoplanin form a complex that is shed into the perivascular stroma and affects the migration of CCR7-positive cells. In the tumor microenvironment, CCL21 acts as a potent chemoattractant for CCR7-positive tumor cells by binding to podoplanin on cancer-associated fibroblasts (CAFs), thereby promoting the stromal invasion of cancer cells [25].

### 2.2. Transmembrane Domain

The transmembrane domain of podoplanin is known to bind CD9 and CD44 [9,27,28]. CD9 is a cell surface protein of the tetraspanin family that has four transmembrane domains. The homophilic interaction between podoplanin and CD9 transmembrane domains 1 and 2 is thought to suppress metastasis by neutralizing podoplanin-mediated platelet aggregation [27]. CD44 is a non-kinase transmembrane glycoprotein adhesion molecule that mediates lymphocyte homing to peripheral lymphoid tissues [29,30]. Since CD44 is expressed in both embryonic stem cells and cancer cell subpopulations, it is also recognized as a molecular marker for cancer stem cells [31]. The podoplanin-CD44 interaction is mediated by transmembrane and cytosolic regions and is negatively modulated by glycosylation of the extracellular domain [32]. In addition to their functions in cancer progression, CD9 and CD44 are differentially expressed by specific lymph node stromal cell populations and can both suppress podoplanin-dependent contractility and contribute toward lymph node expansion during adaptive immune activation [9].

### 2.3. Intracellular Domain

The intracellular domain of podoplanin consists of just nine amino acids; however, ezrin/radixin/moesin (ERM) proteins can bind to the juxtamembrane region, leading to RhoA protein activation and epithelial-mesenchymal transition (EMT) in cancer cells [33,34]. The intracellular domain contains two serine residues that are conserved between mice and humans and can be phosphorylated by protein kinase A (PKA) and cyclin-dependent kinase 5 (CDK5). This can reduce cell motility, presumably by blocking the binding of ERM proteins that activate Rho GTPases and Rho-associated coiled-coil kinase (ROCK) [34,35].

## 3. Podoplanin in Normal Skin

In normal skin, podoplanin is highly expressed in lymphatic endothelial cells, the outer root sheath cells of hair follicle keratinocytes, and the basal cell layer of sebaceous glands, but not in interfollicular epidermis [6]. Interestingly, podoplanin expression correlates with the expression of keratin 15 and CD34, which are putative markers of hair follicle stem cells [10,36]. A recent study demonstrated that podoplanin is expressed in the dermal component of hair follicles in the female scalp as well as in keratinocytes, with its expression decreasing with aging [37]. However, the functional role of podoplanin in dermal papilla cells has not yet been fully elucidated.

Podoplanin expression in lymphatic endothelial cells is thought to prevent the retrograde filling of blood from the circulatory system into the lymphatic system and promote the trafficking of immune cells to lymph nodes [38]. Bianchi et al. generated a postnatal lymphatic-specific podoplanin knockout mouse model in which they observed blood-filled lymph nodes and vessels as well as reduced dendritic cell migration from ear skin to lymph nodes [38]. However, the postnatal deletion of lymphatic-specific podoplanin did not compromise lymph node organization. Together, these results highlight the importance of the interaction between podoplanin in lymphatic cells and CLEC-2 in dendritic cells for dendritic cell migration to lymph nodes.

Yoon et al. investigated the roles of podoplanin in hair follicle growth using a mouse model of hair depilation-induced anagen follicle growth [10]. In wild-type mice, podoplanin expression was absent in keratinocytes in hair follicles during the early- to mid-anagen phase (days 1–5 after depilation), was present during the late-anagen (days 8–12) to catagen (day 18) phase, and then disappeared in the telogen phase. To evaluate the functional effects of podoplanin on hair growth, the authors generated keratin-specific podoplanin deletion mice (K5-Cre;PDPN^flox/flox^ mice) which displayed a thicker hair bulb during the mid-anagen to catagen phase, indicating that podoplanin deletion enhances anagen hair growth. Moreover, hair follicle stem cells isolated from the K5-Cre;PDPN^flox/flox^ mice showed lower focal adhesion and extracellular matrix interaction than the wild-type mice, suggesting that the loss of podoplanin increases the migration of hair follicle stem cells towards the bulb area and promotes anagen hair growth.

## 4. Podoplanin in Inflammatory Skin Diseases

### 4.1. Psoriasis

Psoriasis is a common chronic inflammatory skin disease characterized by scaly erythematous plaques and papules. The histopathology of psoriasis includes acanthosis with regular epidermal elongation, a diminished or absent granular layer, dermal papilla elongation and edema with dilated capillaries, and the infiltration of perivascular lymphocytes and neutrophils into subcorneal epidermis (Munro microabscess) [39]. Thus, keratinocyte proliferation and the inflammatory response appear to play pivotal roles in this disease. Although the pathogenesis of psoriasis is not yet fully understood, recent studies have suggested that disturbances in innate and adaptive cutaneous immune responses, especially the interleukin (IL)-23/Th17 axis and TNF-α signaling, are critically involved in disease development [40,41]. Since prominent epidermal hyperplasia is thought to result from the interaction between keratinocytes and a complex cytokine network due to abnormal T-cell regulation, many effective therapies targeting TNF-α, IL-23, or IL-17 have been developed and used to treat patients with psoriasis [42].

Podoplanin is involved in both keratinocyte proliferation and inflammation during the pathogenesis of psoriasis [43] because it is expressed in keratinocytes and inflammatory cells such as monocytes and Th17 cells [6,43,44]. In psoriasis, podoplanin is expressed in peripheral basal keratinocytes, particularly in highly proliferative lesions lacking a granular layer. Ki-67 expression is also upregulated in these podoplanin-positive basal cells without a granular layer, suggesting that podoplanin affects the migration of basal keratinocytes [44,45,46]. In addition, studies of primary cultured human keratinocytes have revealed that podoplanin is upregulated by transforming growth factor (TGF)-β and interferon (IFN)-γ via the Smad2/3-Smad-4 and JAK-STAT signaling pathways, respectively. Furthermore, IL-22 and IL-6, which play key roles in the pathological mechanism of psoriasis, also induce podoplanin expression via STAT-3 phosphorylation [6].

Recent studies have also indicated that podoplanin contributes toward IL-17 secretion in inflammatory skin diseases [43,47], such as psoriasis. For instance, Noack et al. investigated the role of podoplanin in the interactions between lymphocytes and mesenchymal cells derived from psoriatic skin [43]. High levels of IL-17 secretion were induced when skin fibroblasts were co-cultured directly with activated peripheral blood mononuclear cells (PBMCs), but IL-17 secretion was only slightly upregulated when these cells were co-cultured using a Transwell system to inhibit cell-cell contact. Thus, a direct interaction between PBMCs and fibroblasts is crucial for IL-17 secretion in psoriasis. Moreover, preincubating PBMCs with anti-podoplanin antibodies inhibited the upregulation of IL-17 and the secretion of IL-1β, but not IL-8 or IL-6. Monocyte removal also inhibited IL-17 production, suggesting that monocytes and podoplanin strongly affect IL-17 secretion [43]. Conversely, another study reported that podoplanin is a negative regulator of Th17 inflammation [47]. Nylander et al. demonstrated that podoplanin (+) Th17 cells were induced under classic Th17 polarizing conditions, but did not produce IL-17, and had an upregulated immunosuppressive gene profile (*IL10*, *Ahr*, *Ikzf3*, *FOXO1*, and *FOXO3*) after CD4 T cell activation [47,48]. These regulatory effects of podoplanin were also partially mediated by the CLEC-2/podoplanin interaction, since CLEC-2 significantly increased IL-10 production from polarizing Th17 cell cultures. Together, these observations indicate that the CLEC-2/podoplanin interaction exerts nonpathogenic and possibly regulatory functions in Th17-type inflammation.

### 4.2. Allergic Contact Dermatitis

Allergic contact dermatitis, also known as contact hypersensitivity, is one of the most common skin diseases and is characterized by a delayed hypersensitivity reaction with sensitization and solicitation phases [49]. When the skin comes into contact with haptens, chemicals that induce contact hypersensitivity, antigen-presenting Langerhans cells (LCs) in the epidermis and Langerin-positive dermal dendritic cells (dDCs) work together to initiate sensitization. Once LCs and dDCs capture antigens, they begin to mature and migrate toward draining lymph nodes, where they present the antigens to naïve T cells, leading to sensitization. Recent studies have revealed that podoplanin plays important roles in DC migration and lymph node expansion [9,20,21,50]. Acton et al. demonstrated that CLEC-2 deficiency impairs the entry of DCs into the lymphatic system and their trafficking to lymph nodes, suggesting that the CLEC-2-podoplanin interaction is essential for DC migration to lymph nodes [20]. In addition, the activation of CLEC-2 by podoplanin was found to induce actin cytoskeleton rearrangement and promote the motility of DCs [20]. de Winde et al. investigated the underlying mechanisms and reported that tetraspanin CD37, a membrane-organizing protein, is required for CLEC-2 recruitment to the membrane with podoplanin in order to control CLEC-2-dependent DC migration [9]. In lymph nodes, podoplanin is expressed by fibroblastic reticular cells (FRCs) which form collagen-based reticular networks that act as a scaffold for DCs and T cells and as a conduit for lymph fluid transportation from the subcapsular sinus into the parenchyma of lymph nodes [51,52,53,54]. Podoplanin maintains the microarchitecture of lymph nodes by ensuring the contraction of FRCs under non-inflammatory conditions via RhoA/C and downstream Rho-associated protein kinase activation [21]. However, when DCs migrate into FRC networks during inflammation, binding between CLEC-2 and podoplanin causes rapid podoplanin clustering and prevents RhoA/C activation, thereby relaxing the cytoskeleton, permitting FRC stretching, and resulting in lymph node expansion [21,50].

## 5. Podoplanin in Wound Healing

Wound healing is a dynamic, interactive process that involves various cell types, growth factors, cytokines, and the extracellular matrix [55,56,57]. Wound healing consists of three overlapping phases: inflammation, tissue formation, and tissue remodeling [58]. During inflammation, tissue injury disrupts blood vessels and causes the extravasation of platelet-rich blood constituents that form blood clots and provide a provisional extracellular matrix scaffold for cell migration. The secretion of different cytokines and growth factors attracts and activates macrophages, fibroblasts, vascular endothelial cells, and several bone marrow-derived stem/progenitor cells that promote the formation of granulation tissue. Activated macrophages secrete VEGF-C, which induces lymphangiogenesis [39,40], an important process for maintaining normal tissue pressure by draining lymph fluid from the interstitial space. After blood clots have been replaced by mature, cell-rich granulation tissue, these tissues are rearranged into collagenous scar tissue as part of the tissue remodeling phase, during which re-epithelialization is also promoted.

Podoplanin is thought to contribute toward lymphangiogenesis and re-epithelialization during wound healing [55,59]. Since lymphangiogenesis plays important roles in tissue regeneration and angiogenesis, lymphatic dysfunction can prevent wound healing due to impaired tissue fluid homeostasis [60]. Several studies have indicated that the induction of lymphangiogenesis could be a therapeutic target for impaired wound healing in patients with diabetic ulcers [56,61]. Maruyama et al. demonstrated that podoplanin neutralization by anti-podoplanin antibodies inhibited lymphangiogenesis in a model of ear skin wound healing and inhibited tube formation by human lymphatic endothelial cells in vitro [59]. Thus, podoplanin may be involved in wound healing-associated lymphatic vessel formation.

Podoplanin is expressed very weakly during the inflammatory phase of wound healing, is highly upregulated during the tissue formation-remodeling phase, and then decreases when the wound is completely closed. Thus, podoplanin expression in keratinocytes occurs in parallel with re-epithelialization, suggesting that podoplanin plays a pivotal role in this process [55]. Previously, we demonstrated that silencing podoplanin using siRNA inhibited keratinocyte motility and downregulated RhoA activity, suggesting that podoplanin mediates keratinocyte motility partly via RhoA signaling. Furthermore, podoplanin and E-cadherin expression in keratinocytes were inversely correlated at the wound edge in vivo and E-cadherin was upregulated in podoplanin siRNA-transfected keratinocytes in vitro. Thus, podoplanin downregulation also decreases keratinocyte motility by upregulating E-cadherin, a cell-cell adhesion molecule. During the inflammatory phase, the wound bed is covered with platelet-rich blood clots and is concaved due to limited granulation tissue formation. Early re-epithelialization of this depressed wound bed without abundant granulation tissue is undesirable as this would result in a dented scar. Platelets in blood clots are thought to regulate re-epithelialization during the inflammatory phase in wound healing. For instance, we previously demonstrated that platelets inhibit keratinocyte motility via the direct interaction between podoplanin and CLEC-2 [55]. Consequently, platelets may inhibit re-epithelialization via podoplanin/CLEC-2 during the inflammatory phase and re-epithelialization only proceeds after platelet-rich blood clots have been replaced by mature granulation tissue and podoplanin expression on keratinocytes has been excessively upregulated.

## 6. Podoplanin in Skin Malignancies

Podoplanin is expressed in tumor cells or peritumoral cells and its expression correlates with tumor progression and prognosis in several malignancies of the skin and other organs, including glioblastomas and SCC of the esophagus, head, and neck [62,63,64,65,66,67,68,69,70]. However, podoplanin expression correlates with a good prognosis in cancers such as SCC of the uterus, cervix [71], and lung [72,73,74]. Podoplanin is a critical promoter of tumorigenesis [75,76,77,78], migration [4,25,79], invasion [80,81], EMT [28,80,82], cancer-associated thrombosis [78,83,84,85,86,87], and chemoresistance [88]. The role of podoplanin in skin malignancies has been widely evaluated in melanomas and SCCs, because many cell lines are commercially available and animal models have been established; however, its role in other malignancies has been poorly evaluated, because of the lack of cell lines.

### 6.1. Melanoma

Malignant melanomas are highly aggressive skin cancers with an increasing incidence worldwide. When melanomas occur in mucosal surfaces, the prognosis is very poor [89,90]. Despite remarkable progress in our understanding of tumor immunity and genomic analysis in recent decades, which has led to the development of novel immune checkpoint inhibitor and BRAF/MEK inhibitor therapies for melanoma, the treatment of patients with advanced melanoma remains challenging. Recently, podoplanin has attracted considerable attention as a new therapeutic target for melanoma because both melanoma cells and CAFs express podoplanin [64]. A retrospective analysis of 55 cases of melanoma revealed that podoplanin was expressed in 69.1% of the tumor cells; however, its expression did not correlate with tumor progression [64]. However, podoplanin was expressed in 45.5% of CAFs and was associated with increased tumor thickness and sentinel lymph node metastasis. Furthermore, patients with podoplanin (+) CAFs had a worse prognosis than those with podoplanin (−) CAFs. Despite finding no significant correlation between podoplanin expression and aggressive tumor behavior, several other studies have demonstrated that podoplanin plays a key role in melanoma progression [9,91,92]. For instance, de Winde et al. demonstrated that podoplanin can enhance amoeboid invasion and the dedifferentiation of melanoma cells [9]. Podoplanin expression is increased in metastatic human melanoma cells which have a rounded and contracted morphology; however, podoplanin knockout was found to dramatically alter the morphology of B16F10 murine melanoma cells by increasing their spread and number of protrusions. In vivo, mice injected with podoplanin (+) and podoplanin (−) melanoma cells (1:1 mix) produced tumors containing a higher proportion of podoplanin (+) cells, which were observed beyond the tumor boundary as single rounded cells. These results suggest that podoplanin may either confer a survival advantage or increase the rate of proliferation and promote amoeboid motility [9]. Interestingly, tumors derived from podoplanin (−) cells displayed more pigmentation than podoplanin (+) tumors, suggesting that podoplanin knockout restores the characteristics of non-invasive differentiated melanocytes.

Several preclinical studies have demonstrated that podoplanin could be a therapeutic target in several malignancies, including melanomas [91,92,93,94,95,96,97,98]. For example, lectin extracted from the seeds of the legume tree, *Maackia amurensis* (MASL), has an affinity for O-linked carbohydrate chains containing sialic acid and can bind to podoplanin to inhibit the growth and motility of B16 melanoma cells by inducing caspase-independent necrosis [91]. Since MASL can survive gastrointestinal proteolysis to remain biologically active in the circulatory system, it is able to inhibit melanoma cell migration when fed to mice and inhibit melanoma growth from inoculated B16 melanoma cells by reducing tumor vascularization [91]. Anti-podoplanin monoclonal antibodies are another candidate targeted therapy for melanoma [97,98]. Indeed, the intravenous injection of SZ168 anti-podoplanin monoclonal antibodies was found to significantly suppress pulmonary metastasis in a murine xenograft model inoculated subcutaneously with human melanoma cells [97]. Since melanoma growth is partially regulated via podoplanin-CLEC-2 mediated platelet aggregation, SZ168 is thought to suppress tumor growth by inhibiting the interaction between tumor podoplanin and platelet CLEC-2 [97].

Podoplanin also plays a crucial role in the progression of canine melanomas. Shinada et al. demonstrated that podoplanin is expressed in 80% of canine melanomas and is positively associated with Ki67, a marker of cell proliferation [93]. Moreover, the siRNA-mediated knockdown of podoplanin in canine melanoma cells significantly reduced their migration and invasion capacities. Interestingly, podoplanin knockdown also induced apoptosis and cell cycle arrest at the G2/M phase; however, further studies are required to clarify the underlying molecular mechanism. A cancer-specific mouse–dog chimeric anti-podoplanin antibody, P38B, has also proceeded to phase I/II clinical trial in dogs with melanomas [98] to investigate its safety and anti-tumor effects. In preclinical trials, P38Bf was associated with no adverse events when administered to a healthy dog over two months. In subsequent phase I/II clinical trials, no severe adverse events were observed in three dogs with melanoma treated with P38Bf, and one dog exhibited stable disease [98]. Thus, targeted therapy with anti-podoplanin antibodies has therapeutic potential against melanoma.

### 6.2. Cutaneous SCC

Cutaneous SCC is the second most common skin malignancy arising from epidermal keratinocytes and several studies have evaluated the association between podoplanin expression and the clinical progression of this disease [66,99,100,101]. Most studies have suggested that tumor podoplanin expression correlates positively with aggressive tumor behaviors such as lymph node metastasis, local aggression, and survival rate. Although podoplanin is also expressed in stromal cells in SCC, its function has not been fully elucidated [102].

During SCC development and progression, podoplanin is thought to mediate tumorigenesis, EMT, invadopodia, and cell migration [4,28,33,79,80,103,104]. Scholl et al. demonstrated that the ectopic podoplanin expression in keratinocytes induced cell surface extensions, increased motility, downregulated epithelial markers (basal keratin K14), and upregulated mesenchymal markers (vimentin), suggesting that podoplanin induces EMT. Furthermore, ectopic podoplanin expression induced tumorigenic and metastatic properties [104]. However, chemically-induced tumor development does not decrease in mice with a specific podoplanin deletion in the epidermal keratinocytes, indicating that podoplanin is dispensable in skin carcinogenesis [105].

Invadopodia are actin-rich cell membrane protrusions found in invasive cancer cells [106] that can penetrate the basement membrane by degrading the extracellular matrix during cancer invasion and metastasis. Podoplanin downregulation in SCC cells can decrease invadopodia stability and impair extracellular matrix degradation [80]. Interestingly, the podoplanin-CD44 interaction has been shown to play a pivotal role in SCC cell migration, with their colocalization on cell surface protrusions mediating the directional motility of SCC cells [28]. CD44v3-10, CD44v6-10, and CD44v8-10 are the major CD44 variant isoforms co-expressed with the standard CD44 isoform and podoplanin in SCC cells, suggesting that both the standard and variant isoforms may interact with podoplanin during the pathogenesis of SCC [32].

### 6.3. Extramammary Paget’s Disease (EMPD)

EMPD is a rare skin cancer with an extremely poor prognosis in patients with metastasis. In in situ EMPD, tumor cells are located in the epidermis just above the basal cell layers, whereas tumor cells penetrate the basal cell layers into the dermis in invasive EMPD. Previously, we reported that podoplanin expression in peritumoral basal keratinocytes, but not in tumor cells, is associated with tumor thickness and dermal invasion [65]. The downregulation of E-cadherin in podoplanin (+) keratinocytes may contribute toward the dermal penetration of tumor cells by decreasing cell adhesion between basal cells to create gaps for invasion. Moreover, podoplanin (+) keratinocytes possess invadopodia, which may assist dermal invasion by degrading the extracellular matrix in basal cell layers. Furthermore, EMPD cells are positive for TGF-β expression, suggesting that tumor cells control peritumoral keratinocytes to assist tumor invasion by upregulating podoplanin via TGF-β [65]. The lack of a useful model has greatly limited our ability to directly evaluate EMPD pathogenesis; however, a method for the 3D culture of primary EMPD cells was recently established for the first time and allowed the successful generation of xenograft murine models [107]. Consequently, the mechanisms via which podoplanin mediates tumor progression in EMPD could soon be clarified in vivo.

### 6.4. Mycosis Fungoides and Sezary Syndrome

Mycosis fungoides is the most common cutaneous T-cell lymphoma; it clinically manifests as patches, plaques, tumors, and erythroderma [108]. The disease exhibits slow progression and the clinical course is stable; however, in some cases, it manifests aggressive behavior and disseminates to the lymph nodes and internal organs.

In mycosis fungoides, podoplanin is expressed in the basal cell layer of the epidermis, the malignant lymphocytes in epidermis and dermis, and the lymphatic vessels in dermis [69,109]. There is a significant positive correlation between the intensity of podoplanin expression in the basal cell layer of the epidermis, malignant lymphocytes, and lymphatic vessels and the TNMB staging of mycosis fungoides [69]. Increased expression of VEGF-C and lymphatic vascularization are observed in highly infiltrated and extensive cutaneous lesions, indicating tumor-induced lymphangiogenesis; this contributes to the progression of mycosis fungoides [109]. There is a positive correlation between the number of podoplanin-positive vessels and disease progression in Sézary syndrome, which is considered the most severe cutaneous lymphoma [110]. Therefore, podoplanin could be used as a predictive marker for the aggressive behavior in mycosis fungoides and Sézary syndrome.

## 7. Conclusions

The roles of podoplanin in skin diseases are summarized in Table 1. In inflammatory diseases, podoplanin is thought to control dendritic cell migration to lymph nodes and to regulate Th17-type immune responses. In psoriasis, the effects of podoplanin on Th17 inflammation are controversial and further studies are required to elucidate the podoplanin-mediated cellular pathways that affect immune responses and, thus, the functional roles of podoplanin during the pathogenesis of psoriasis. The roles of podoplanin in wound healing are much clearer than that in the immune response; podoplanin promotes keratinocyte migration. Therefore, podoplanin could have therapeutic potential in patients with impaired wound healing (e.g., diabetic foot ulcers) when upregulated in the keratinocytes at the wound edge. However, the increased risk of carcinogenesis due to podoplanin upregulation should be considered, because a chronic wound in itself is a risk factor for skin cancers. The role of podoplanin has also been elucidated in various skin cancers and the targeted therapy of melanomas using anti-podoplanin antibodies has already been evaluated in preclinical trials. Future studies to elucidate the roles of podoplanin during cancer invasion and metastasis could therefore help to establish new podoplanin-based anti-cancer therapies.

## Figures and Tables

**Table 1:** Role of podoplanin-expressing cells in skin diseases.

Disease	Cell Type	Role
Psoriasis	Keratinocytes	Epidermal elongation
	Th17 cells	IL-17 ↓
	Monocytes	IL-17 ↑
Allergic contact dermatitis	LECs	Mediate migration of DCs and LCs
	FRCs	Lymph node expansion
Wound healing	LECs	Lymphangiogenesis
	Keratinocytes	Re-epithelialization
Melanoma	CAFs	Biomarker
	Tumor cells	amoeboid invasion and dedifferentiation
cSCC	Tumor cells	EMT, invadopodia
EMPD	Keratinocytes	Invadopodia
Mycosis fungoides	Basal cell layer Malignant lymphocytes LECs	lymphangiogenesis
Sézary syndrome	LECs	lymphangiogenesis

LECs: lymphatic endothelial cells. DCs: dendritic cells. LCs: Langerhans cells. FRCs: fibroblastic reticular cells. CAFs: cancer-associated fibroblasts. cSCC: cutaneous squamous cell carcinoma. EMT: epithelial-mesenchymal transition. EMPD: extramammary Paget’s disease. ↑: upregulation. ↓: downregulation.
